# Supplementary material for: Transcriptional Inhibition of AGPAT2 Induces Abnormal Lipid Metabolism and Oxidative Stress in the Liver of Nile Tilapia Oreochromis niloticus
Source: Antioxidants (Basel). 2023 Mar 12;12(3):700. doi: 10.3390/antiox12030700 (PMC10045202; doi:10.3390/antiox12030700)
Supplement: Supplementary file 1 [file antioxidants-12-00700-s001.zip › Table S1.pdf]

Table S1. Overview of reads for mRNA-seq and quality filtering.

Sample	Raw Data		Valid Data		Valid Ratio	Q20%	Q30%	GC%
	Read	Base	Read	Base				
AT2_1	36673256	5.50G	34806726	5.22G	94.91	99.95	98.67	46.50
AT2_2	43542874	6.53G	40051252	6.01G	91.98	99.95	98.66	46
AT2_3	49943870	7.49G	46186856	6.93G	92.48	99.95	98.58	46.50
CON_1	45254716	6.79G	38107378	5.72G	84.21	99.95	98.54	45
CON_2	45791028	6.87G	42881204	6.43G	93.65	99.95	98.62	46.50
CON_3	53528600	8.03G	51053306	7.66G	95.38	99.75	97.05	47
